# Incidence of blood culture-related sepsis in neonates and antibiotics sensitivity of implicated organisms in a secondary healthcare facility in Ghana

**DOI:** 10.4314/gmj.v57i2.8

**Published:** 2023-06

**Authors:** Francis K. Wuni, Margaret W. Kukeba, Kwashie S. N. Dzotsi, Abu Osman, Patrick Atobrah, Rasheed Ofosu-Poku

**Affiliations:** 1 Newborn Care Unit, Upper East Regional Hospital, Bolgatanga, Ghana; 2 CK Tedam University for Technology and Applied Sciences, Navrongo, Upper East Region, Ghana; 3 Microbiology Laboratory. Upper East Regional Hospital, Bolgatanga, Ghana; 4 Directorate of Family Medicine, Komfo Anokye Teaching Hospital, Kumasi, Ghana

**Keywords:** neonates, neonatal sepsis, antibiotics

## Abstract

**Objective:**

We determined the incidence of blood culture-related sepsis, causative bacteria, and antibiotics sensitivity among newborn babies with suggestive signs of sepsis admitted at the Upper East Regional Hospital in Bolgatanga, Ghana.

**Design:**

Prospective cross-sectional study

**Setting:**

Newborn Care Unit of the Upper East Regional Hospital, Bolgatanga

**Participants:**

Neonates admitted to the Newborn Care Unit from August 2019 to August 2020 with signs of sepsis

**Main outcome measures:**

Organisms isolated from blood cultures and sensitivity of isolated organisms to antibiotics.

**Results:**

The study included two hundred and seventy-six (276) patients. Laboratory confirmed sepsis was 13.4% (37/276). Early onset sepsis was 3.3% (9/276), while late-onset sepsis was 10.1% (28/276). The most common clinical signs associated with positive culture cases were temperature instability (35.5%), poor feeding (14.5%), neonatal jaundice (11.3%), vomiting (9.7%), and respiratory distress (8.1%). *Staphylococcus aureus* and *Staphylococcus epidermidis* were the most common bacterial isolates (46% and 32%, respectively). There was no relationship between independent variables and blood culture confirmed sepsis. Antibiotics to which isolates were most resistant included flucloxacillin 4/4, penicillin 14/15, ampicillin 16/18, and tetracycline 23/28. Bacterial isolates were most sensitive to amikacin 16/16, levofloxacin 5/5, erythromycin 8/8, cefazolin 7/8, and ciprofloxacin 18/24.

**Conclusion:**

Late-onset sepsis is a common sepsis category, and the implicated microorganisms are resistant to commonly prescribed antibiotics.

**Funding:**

This work was funded by Upper East Regional Hospital, Bolgatanga.

## Introduction

Neonatal sepsis is the leading cause of admission in newborn care units and the leading cause of neonatal death in Ghana.^1^ It increases the period of hospital stay, causes distress to the newborn, reduces their initial growth and development, and burdens parents physically and financially.^2^

The World Health Organization recommends using ampicillin (or penicillin) and gentamycin as the first-line treatment for neonatal sepsis.^3^ However, there is evidence of high levels of resistance to these recommended antibiotics.^4^,^5^ Many studies have also revealed high resistance to other drugs, especially cephalosporins.^4^,^6^ Such resistance, according to a study conducted on the impact of antibiotics resistance, is associated with a high cost of treatment and delayed recovery and development of the newborn in developing countries.^7^ To prevent the implications of resistance of organisms to antibiotics, empiric antibiotics therapy is required to effectively manage sepsis in newborn babies.^4^ The latter requires the identification of the most common causative organisms in a particular setting.^5^,^8^,^9^

The causative agents differ from time to time and region to region, requiring periodic specific identification of the causative organisms of neonatal sepsis.^10^ While a study in 2016 in Ghana at the Korle-Bu Teaching Hospital found *Staphylococcus aureus* as the most common organism responsible for neonatal sepsis^4^, a similar study within the same period at the Lagos University Teaching Hospital in Nigeria found *Klebsiella pneumoniae* as the predominant organism.^11^

The need for antibiotic sensitivity data to guide the choice of antibiotics for managing neonatal sepsis is essential for low-income settings like northern Ghana, where the cost of laboratory services, including blood culture and sensitivity tests, exceeds the income of most parents.

This study contributes to data on the incidence of neonatal sepsis, the specific causative organisms and the most effective antibiotics in their management. Consequently, this will promote and ensure appropriate empiric antibiotics therapy in the management of neonatal sepsis in the facility.

## Methods

### Study setting

The study was conducted at the Newborn Care Unit of the Upper East Regional Hospital. The Hospital is a referral centre for the region and surrounding districts in the North East Region of Ghana. Neonates from Yagaba and Walewale and their surrounding villages in the Mamprugu Moagduri and West Mamprusi Districts, respectively, are referred to this hospital. It has a bed complement of 220 serving a catchment population of about 1,540,049. The Newborn Care Unit has twenty cots, four functioning incubators, and five beds at the Kangaroo Mother Care (KMC) unit. Annually, the unit admits an average of thousand and eighty-two (1082) neonates and those presenting with signs of neonatal sepsis account for nearly 59.7%.

### Study design

A prospective cross-sectional survey of neonates admitted to the Newborn Care Unit of the Upper East Regional Hospital was used. Neonates admitted with clinical signs of sepsis who met the inclusion criteria were all selected for the study.

### Study population

The study population comprised preterm and term neonates aged 0-28 days of life admitted from August 2019 to August 2020 at the Newborn Care Unit of the Upper East Regional Hospital. Neonates admitted into the unit came from the labour units of the hospital and its referring facilities, and some from their various homes, having been discharged after delivery.

Neonates were enrolled in the study on the basis of selected clinical signs of possible serious bacterial infections as used in previous studies.^5^,^6^,^8^ All neonates on admission during the study period were observed for these clinical signs of sepsis. Neonates with any one or more of the following possible signs of sepsis were included in the study: temperature >38.5°C or < 36°C, poor feeding, vomiting, respiratory distress, excessive crying, irritability, lethargy, seizures, hypoglycaemia, hyperglycemia, and neonatal jaundice. Neonates with congenital anomalies and metabolic disorders who had undergone surgery or who had received antibiotics were excluded from the study. Three hundred and twelve (312) neonates met the inclusion criteria, and two hundred and seven six (276) were selected for the study. The remaining 11.5% (36) of eligible patients were not included in the study because of the following: parents of nine (9) babies were not available to sign the study consent form, we could not obtain the blood sample in seventeen (17) neonates, and in ten (10) neonates, blood culture bottles were not available for the investigators to collect the specimen. Of the 276 neonates included in the study, 144 were born at the Regional Hospital, whiles the remaining 132 were outborn/referred. Those that were admitted at age < 3 days of life were 134, 3-7 days were 70 and 8-28 days of life were 72.

### Specimen collection and processing

The blood sample was taken from the peripheral veins of each participant after disinfecting the skin at the venipuncture site with a 70% alcohol-based skin swab. The laboratory personnel or the ward staff taking the sample had to wait for the site to dry before the venipuncture. A cannula size 24-gauge was used to collect an estimated 2-3mls of blood from each neonate into a paediatrics blood culture bottle containing a broth prepared in the laboratory using Brain Heart Infusion manufactured by Biomark Laboratories to support bacterial growth. Every batch of Brain Heart Infusion broth prepared was controlled with a positive and negative specimen to ensure they functioned as expected. These were then stored in a refrigerator whose temperature was monitored twice daily to ensure that reagents were stored within the required temperature. Each sample was submitted within two hours to the microbiology laboratory for processing. This has been supported by a prior study recommending processing of the blood specimens at the microbiology laboratory within two hours after collecting the specimen into culture bottles^12^. All samples were incubated under 37°C and examined daily for visible microbial growth using an aseptic technique to avoid contamination. The broth in the culture bottles was further subcultured onto blood agar (BA), chocolate agar (CA), and MacConkey agar plates. The final subculture for each sample was performed on day seven.

This is because manual blood culture is often incubated for 7 days. All isolates were tested for antimicrobial sensitivity using the modified Kirby-Bauer method.

Culture bottles with possible growth were stored at a temperature of -30°C for a period of six months so that investigators could refer to them from time to time for crosschecking. Ethics approval for this study was granted by Navrongo Health Research Centre Institutional Review Board (ID: NHRCIRB352).

### Data collection and analysis

Participants' Demographic data, such as sex, weight and days of life, were collected from their medical records. The gestational age of each participant was determined using either scan results or the mother's last menstrual period recorded by midwives. The investigators reviewed some of the patients' clinical features to establish severe infections and rule out contaminants. The demographic data and clinical signs of neonatal sepsis were obtained using a designed data collection tool. The data were entered into EpiData software version 3.02. Data management and analysis were conducted using STATA software version 15.0. The incidence of neonatal sepsis was calculated as the number of episodes of sepsis with positive blood culture results per the total number of participants in the study multiplied by 100. Descriptive analysis was conducted on continuous variables (means, standard deviation) and categorical variables (simple tabulation, cross-tabulation). Results were reported in tables and charts in percentages and frequency. Bivariate analysis using Fisher's exact test was conducted to assess the relationship between blood culture-confirmed sepsis and the sex of neonates, place of delivery, type of delivery, and maturity.

## Results

Two hundred and seventy-six (276) neonates with a mean age of 5.6 days and median age of 8 days were enrolled in the study. The minimum number of days of life was zero (0), and the maximum was twenty-six (26). Males were 152 (55.1%), while females were 124 (44.9%). The participants were categorised into four groups by their weight at birth: Extreme low birth weight (< 1000g) (0%), very low birth weight (1000g-1500g) 21 (7.6%), low birth weight (1500g-2500g) 76 (27.5%), and normal birth weight (>2500g) 179 (64.9%). Preterm babies were 60 (21.7%), and term babies were 216 (78.3%). All variables were found to have no significant relationship with blood culture-confirmed sepsis. Table 1 illustrates the demographic characteristics of the neonates involved in the study.

**Table 1:** Demographic characteristics of neonates investigated for sepsis

Characteristics	Frequency, n (%)
**Sex**	
**Male**	152 (55.1)
**Female**	124 (44.9)
**Maturity**	
**Preterm**	60 (21.7)
**Term**	216 (78.3)
**Place of delivery**	
**Health facility**	267 (96.7)
**Home**	9 (3.3)
**Type of delivery**	
**Normal delivery**	216 (78.3)
**Caesarean section**	60 (21.7)

### Clinical signs of neonatal sepsis at presentation

A total of ten (10) suggestive signs of neonatal sepsis were used to enrol participants for this study. These were temperature >38.5°C or < 36.0 °C, poor feeding, vomiting, respiratory distress, excessive cry, irritability, lethargy, seizures, hypoglycaemia, and neonatal jaundice. Most of the babies presented more than one clinical sign. In all, participants presented 414 clinical signs of neonatal sepsis.

**Figure 1:**
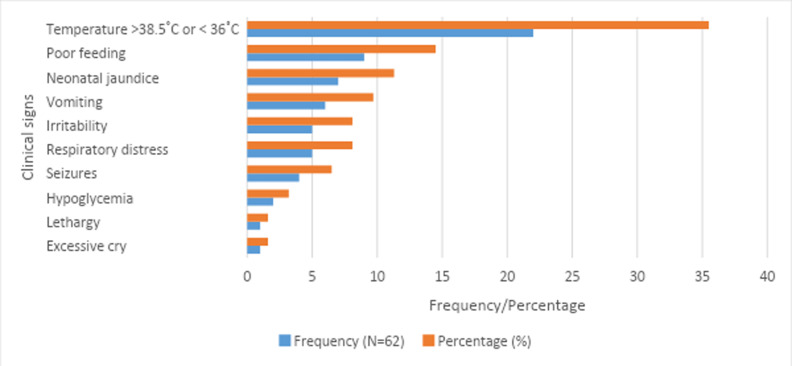
Clinical signs in neonates with proven blood culture positive

The most common clinical signs presented were abnormal body temperature (either pyrexia or hypothermia) (81, 19.6%), neonatal jaundice (57, 13.8%), poor feeding (55, 13.3%), respiratory distress (43, 10.4%) and lethargy (43, 10.4%). The commonest clinical sign presented by neonates with positive blood culture in the study was temperature instability (35.5%). Poor feeding (14.5%), neonatal jaundice (11.3%), vomiting (9.7%) and respiratory distress (8.1%), were the other clinical signs manifested by positive cases. Figure 1 has details of positive cases and the clinical features manifested.

### Neonatal sepsis and bacterial isolates

**Figure 2:**
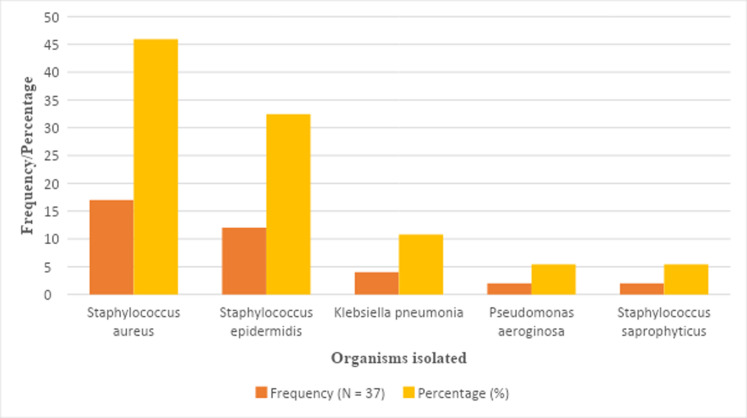
Bacterial isolates from blood culture

Thirty-seven of the 267 newborns in the study had blood cultures positive for bacteria indicating a neonatal sepsis incidence rate of 13.4%. This is the incidence rate of culture proven sepsis among neonates with one or more of the stated clinical features. The percentage of neonates with blood culture positive results who presented with clinical signs of sepsis within the first 72 hours of life (early-onset sepsis) was 24.3% (9/37), whiles those after 72 hours of life (late-onset sepsis) was 75.7% (28/37). Five organisms were isolated from the 37 babies with positive blood cultures, as illustrated in Figure 2. *Staphylococcus aureus* was the most isolated organism, accounting for (17, 45.95%). This was followed by *Staphylococcus epidermidis* (12, 32.43%), *Klebsiella pneumoniae* (4, 10.81%), *Pseudomonas aeruginosa* (2, 5.41%) and *Staphylococcus saprophyticus* (2, 5.41%). The clinical features of the patients with *Staphylococcus saprophyticus* were reviewed to establish severe infections and to rule out issues of contaminates.

### Sensitivity of causative organisms to various antibiotics

Bacterial isolates were tested for antibiotic sensitivity using antibiotic sensitivity discs available at the microbiology unit each time. We did not specify the choice of antimicrobial drugs whose susceptibility testing was to be conducted as part of the study design. This led to the differences in the number of tests done for each antimicrobial drug in this study. Antibiotics with high resistance in this study included flucloxacillin 4/4, penicillin 14/15, ampicillin 16/18, and tetracycline 23/28. However, those with high sensitivity were amikacin 16/16, levofloxacin 5/5, erythromycin 8/8, cefazolin 7/8, and ciprofloxacin 18/24. Table 2 details the antibiotic resistance and sensitivity pattern and the number tested per antibiotic.

**Table 2:** Pattern of antibiotic sensitivity and resistance

Antibiotic	Number of patients tested	Sensitivity N (%)	Resistance N (%)
**Ampicillin**	18	2 (11.1)	16 (88.9)
**Amikacin**	16	16 (100)	-
**Gentamycin**	20	14 (70.0)	6 (30.0)
**Ciprofloxacin**	24	18 (75.0)	6 (25.0)
**Cefuroxime**	23	10 (43.5)	13 (56.5)
**Penicillin**	15	1 (6.7)	14 (93.3)
**Ceftriaxone**	9	2 (22.2)	7 (77.8)
**Erythromycin**	8	8 (100)	-
**Cefotaxime**	5	-	5 (100)
**Flucloxacillin**	4	-	4 (100)
**Tetracycline**	28	5 (17.9)	23 (82.1)
**Co-trimoxazole**	26	7 (26.9)	19 (73.1)
**Amoxicillin**	10	4 (40.0)	6 (60.0)
**Chloramphenicol**	15	8 (53.3)	7 (46.7)
**Cefazolin**	8	7 (87.5)	1 (12.5)
**Levofloxacin**	5	5 (100)	-
**Cephalexin**	9	7 (77.8)	2 (22.2)
**Azithromycin**	7	2 (28.6)	5 (71.4)
**Norfloxacin**	2	1 (50.0)	1 (50.0)

## Discussion

Resistance to antibiotics in neonates has become a global concern, and locally generated evidence is recommended for effective management of neonatal sepsis. This study sought to determine the incidence of neonatal sepsis, the causative organisms, and their sensitivity to various antibiotics.

The incidence of neonatal sepsis in the newborn care unit was 13.4% (37/276 participants) among patients with at least one clinical feature of sepsis. This current study reported a lower incidence than prior studies in Africa^4^,^8^,^13^ This may be attributed to variations in the study settings. However, a recent study in Bhutan reported a lower incidence (1.9%) of laboratory confirmed sepsis in neonates.^14^ Although the incidence appears to be generally low, neonatal sepsis can have dire implications for neonates, their parents, and the health care system. Even though many clinical signs could suggest neonatal sepsis, body temperature extremes were the most common clinical signs for presumed sepsis (19.6%) and laboratory confirmed sepsis (35.5%).

These findings are inconsistent with other studies that found respiratory distress as the common clinical sign for presumed and laboratory confirmed neonatal sepsis.^6^,^8^,^15^ Considering the wide range of clinical features that could be associated with neonatal sepsis, investigating further to determine the features that are peculiar to specific geographical areas is crucial in contributing to the initiation of an early treatment since access to laboratory facilities to confirm that the diagnosis is lacking in most developing settings. Moreover, to ensure a balance between antibiotic misuse and essential use, a good understanding of clinical features will ensure that neonates receive early treatment without the fear of irrational use.

The study findings showed that late-onset sepsis was 10.1%, whiles early-onset sepsis was 24.3%. Most studies have also reported high rates of late-onset neonatal sepsis.^6^,^8^,^16^ Although speculative, considering that most of the neonates were delivered at health facilities, some reporting from home having been discharged after delivery suggests multiple sources of infection. Perhaps, an investigation into the sources of neonatal infections in this study setting would help place preventive strategies appropriately.

*Staphylococcus Aureus* was the most common organism (46.0%). Other studies have reported the same organism as the commonest bacterial isolate in the aetiology of neonatal sepsis.^5^,^13^,^17^ *Klebsiella pneumoniae* and *Enterobacter spp* were, however, reported as the predominant organisms responsible for neonatal sepsis in other studies.^11^,^18^,^19^ The other organisms implicated, as shown in the results, also appear to differ from the findings of other studies.^8^,^11^,^13^ This suggests that the recommendation for health facilities to conduct context-specific investigations on common isolates implicated in the aetiology of neonatal sepsis remains critical.

The sensitivity of isolated bacterial pathogens to antibiotics showed that most neonates were not receiving antibiotics that were likely to be effective for their infections. Amikacin, levofloxacin, erythromycin, and cefazolin were some effective antibiotics against the isolated bacteria. However, levofloxacin and cefazolin were never used, whereas amikacin and erythromycin were used minimally in the study setting. Indeed, apart from amikacin, none of the antibiotics identified to be effective against the common pathogens in this study is part of the current protocol for managing sepsis in the setting. The first and second-line antibiotics used in managing neonatal sepsis in the study setting are ampicillin or flucloxacillin, gentamycin, cefotaxime, and gentamycin. Meanwhile, the isolates were resistant to ampicillin, flucloxacillin, and cefotaxime. As indicated, other studies have also reported on the considerable resistance of bacterial pathogens to ampicillin and other commonly used antibiotics.^5^,^8^,^20^ The investigators did not acquire antibiotics disk containing recommended antibiotics for the study. We relied on the available antibiotics disks for blood culture at the microbiology unit, and some of these disks did not contain commonly used antibiotics in neonatal sepsis. As a result, antibiotics such as flucloxacillin and cefotaxime were tested in only 4 and 5 isolated organisms, respectively, limiting the recommendation on using these drugs in this study.

The findings of this study show that most of the commonly used drugs in managing neonatal sepsis in a unit over time may not be effective.

Identifying the common causative organisms and their sensitivity to antibiotics is essential to effectively treat newborn sepsis. However, to ensure that neonates are promptly treated, short-time blood culture technology may be required for fast testing of neonates' blood to identify these common causative organisms and effective antibiotics.

### Limitations

This study focused only on blood culture-related sepsis. Cerebrospinal fluids (CSF) samples were not taken for smear microscopy, culture, and sensitivity. Urine samples were also not collected for urinalysis, culture and sensitivity. Meanwhile, microorganisms can equally be isolated from CSF, urine, and other sites when neonates have sepsis. This limits the generalisation of the study findings.

## Conclusion

The findings of this study give an insight into the incidence of blood culture-related sepsis, causative organisms and the sensitivity patterns of microbial pathogens. While the recovery of bacterial isolates is necessary for diagnosing neonatal sepsis, the availability of antimicrobial sensitivity data is also needed in choosing antibiotics to effectively manage sepsis in newborns. The findings also pose a new challenge in the care of neonates with suggested signs of sepsis. There is a need to get antimicrobial sensitivity data from urine culture-related sepsis and cerebrospinal fluids culture-related sepsis for optimal recovery of newborn babies with sepsis.
